# Hepatopulmonary Syndrome: A Comprehensive Review

**DOI:** 10.7759/cureus.65204

**Published:** 2024-07-23

**Authors:** Abeer Qasim, Abhilasha Jyala, Shitij Shrivastava, Nishant Allena, Haider Ghazanfar, Vedangkumar Bhatt, Husnain R Ali, Trupti Vakde, Harish Patel

**Affiliations:** 1 Internal Medicine, BronxCare Health System, New York, USA; 2 Pulmonary Medicine, BronxCare Health System, New York, USA; 3 Medicine and Surgery, BronxCare Health System, New York, USA; 4 Medicine, American University of the Caribbean School of Medicine, Miramar, USA; 5 Pulmonary and Critical Care, BronxCare Health System, New York, USA

**Keywords:** model for end-stage liver disease (meld), arterial blood gas, alveolar gas exchange, review article, hepatopulmonary syndrome

## Abstract

Hepatopulmonary syndrome (HPS) is defined by abnormally dilated blood vessels and shunts within the lungs, leading to impaired oxygen exchange. This condition results from intricate interactions between the liver, the gastrointestinal system, and the lungs. This complex system primarily affects pulmonary endothelial, immunomodulatory, and respiratory epithelial cells. Consequently, this contributes to pathological pulmonary changes characteristic of HPS. A classification system based on the severity of oxygen deficiency has been proposed for grading the physiological dysfunction of HPS. Contrast-enhanced echocardiography is considered the primary radiological evaluation for identifying abnormal blood vessel dilations within the lungs, which, combined with an elevated alveolar-arterial gradient, is essential for making the diagnosis. Liver transplantation is the sole effective definitive treatment that can reverse the course of the condition. Despite often being symptomless, HPS carries a significant risk of mortality before transplantation, regardless of the severity of liver disease. Meanwhile, there is varying data regarding survival rates following liver transplantation. The adoption of the model for end-stage liver disease (MELD) standard exception policy has notably improved the results for individuals with HPS compared to the period before MELD was introduced. This review offers a summary of the present understanding, highlighting recent advancements in the diagnosis and treatment of HPS. Furthermore, it aims to augment comprehension of the condition's fundamental mechanisms through insights derived from experimental models and translational research.

## Introduction and background

Hepatopulmonary syndrome (HPS) is characterized by low blood oxygen levels along with portal hypertension, primarily caused by the dilation of small blood vessels within the lungs. HPS is clinically characterized by platypnea-orthodeoxia, which is the shortness of breath and hypoxemia that worsens in an upright position [1]. HPS can be characterized by an elevated alveolar-arterial oxygen gradient (P(A-a)O2) on ambient air, which typically exceeds 15 mmHg, or in cases of patients aged over 64 years, it can be greater than 20 mmHg. This condition may occur with or without hypoxemia and is a consequence of abnormal lung blood vessel dilation in individuals who have liver dysfunction or portal hypertension [2]. The defining characteristic of pulmonary vascular alterations in HPS involves the dilation of blood vessels at the level just before the capillaries and within the capillaries themselves, along with the development of intrapulmonary shunting between arteries and veins. These changes result in the redirection of deoxygenated blood flow from the right side of the heart to the left, causing a mismatch between air ventilation and blood circulation, and limitations in the exchange of gases. In medical literature, the reported prevalence of HPS in cirrhotic patients ranges between 4% and 32% [3].

## Review

HPS is a term coined by Kennedy and Knudson in 1977. However, the clinical symptoms have been described initially back in 1884 by Fluckiger, who assessed a woman presenting with cirrhosis, cyanosis, and clubbing [4,5]. The prevalence ranged from 4% to 47% in patients with chronic liver disease [3]. The average prevalence among multiple studies is about 25% when the diagnostic tools are pulmonary function tests, transthoracic contrast echocardiography (TTCE), and blood gas analysis [3,6]. It should be noted these results are analyses of data collected from liver transplant centers with limited studies carried out in alternate settings.

Additionally, the frequent changes in diagnostic threshold values contribute to the extensive variability in the prevalence rates of HPS patients [3]. Studies have indicated that among individuals who are eligible for a liver transplant, about 13-80% have intrapulmonary vascular dilatations irrespective of the presence of arterial oxygenation abnormalities [7]. While individuals with liver disease have a higher susceptibility to developing HPS, a study conducted by Pascasio et al. reveals that the majority of these patients (92.6%) are in the mild or moderate stages. Additionally, the study found no discernible connection between the severity of liver disease and HPS [8]. Following the identification of the alveolar-arterial gradient as the ideal sensitive indicator for HPS, there has been a surge in the detection of the condition as an asymptomatic patient is now also being screened [9,10]. In a retrospective analysis conducted by Dahiya et al., utilizing the nationwide inpatient sample, it was observed that hospitalizations for HPS rose from 1,565 in 2012 to 2,495 in 2018. The average age of patients ranged from 55.8 to 58.1 years during this period. Furthermore, the study indicated a marginal decline in female hospitalizations, decreasing from 50.5% in 2012 to 47.3% in 2018 [11].

Additionally, a study conducted by Fallon et al. indicated a higher prevalence of this condition in the non-Hispanic White population when compared to Hispanic, non-Hispanic Black, and other specified racial or ethnic groups. The primary causes of liver disease in a majority of HPS patients were identified as alcohol use and/or hepatitis C infection. Interestingly, studies show patients with HPS are infrequently current or past smokers [12]. HPS is rare in children; studies have shown it is most prevalent among those with biliary atresia (3-20%) and portal vein thrombosis (0.5%) [13]. This condition is known to have limited data regarding its incidence and prevalence due to its changing diagnostic criteria and frequent occurrences with comorbidities causing an oversight.

Pathogenesis

HPS is typically associated with advanced liver disease, such as cirrhosis, portal hypertension, or other severe hepatic conditions [14]. The primary cause of HPS is believed to be pulmonary vascular dilatation, which arises from an imbalance between substances that promote vasodilation and those that induce vasoconstriction. The liver undergoes structural changes in cirrhosis, and the normal architecture is disrupted. This results in heightened resistance to the flow of blood through the liver, leading to portal hypertension. Consequently, portal hypertension diminishes blood circulation to the intestines, leading to an increase in gram-negative bacteria and the movement of endotoxins into the bloodstream. This occurrence triggers the release of vasoactive agents like tumor necrosis factor-alpha (TNF-α), carbon monoxide (CO) derived from heme oxygenase (HO), and nitric oxide (NO) [15].

In cirrhotic patients with HPS, exhaled NO levels increase compared to cirrhotic control patients. Plasma endothelin-1 (ET-1) levels also rise in patients with cirrhosis and intrapulmonary vascular dilatation. ET-1 induces NO-related vasodilation by activating endothelin B receptors (ETBR) on endothelial cells. Also, the increased phagocytosis of bacterial endotoxin in the lungs triggers the activation of inducible NO synthase (iNOS), further contributing to an increase in NO production. Pulmonary angiogenesis in HPS is stimulated by bacterial translocation and monocyte accumulation with genetic factors also playing a role in its regulation [16]. Cytokines and chemokines are believed to be influential in this process. The prevalence of this condition is higher in individuals possessing the monocyte chemoattractant protein-1 (MCP-1) 2518G gene [14]. In contrast, it occurs less frequently in individuals with the endothelial NO synthase (eNOS) 298Asp allele and those carrying the eNOS 298Asp variant. These findings imply that the endothelial eNOS 298Asp polymorphism may protect against the onset of HPS in patients with cirrhosis. Furthermore, the G allele is potentially linked to elevated MCP-1 expression during certain inflammatory conditions. The impact of the G allele seems to be dosage-dependent, with cells from individuals homozygous for G at position -2518 exhibiting higher MCP-1 production than G/A heterozygotes. Elevated levels of MCP-1, an inflammation marker observed in HPS, suggest the involvement of inflammation in the development of pulmonary shunts. Additionally, macrophages produce heme oxygenase-1 (HO-1), increasing CO production and contributing to vasodilation. CO is generated through the breakdown of heme by heme-oxygenase [17]. The heightened synthesis of NO and CO, potent vasodilators, is a pivotal factor in inducing pulmonary vasodilation. Additionally, the activation of the vascular endothelial growth factor (VEGF) by macrophages, monocytes, and TNF-α contributes to enhanced angiogenesis in the pulmonary vasculature. While various mediators like somatostatin analog (octreotide), glucagon, prostacyclin, angiotensin-2, vasoactive intestinal peptide, calcitonin, substance P, atrial natriuretic factor, and platelet-activating factor have been considered in the development of vascular alterations in HPS, no conclusive connection has been established between these mediators and the observed vascular dilatation [18].

The vasodilation and angiogenesis result in the formation of arteriovenous (AV) shunts in the pulmonary vasculature, causing a mismatch between ventilation and perfusion. In HPS, pulmonary capillaries exhibit dilation ranging from 15 to 500 mm, contrasting with the normal 8-15 mm diameter [19]. The pulmonary vasodilation decreases the transit time for blood cells, allowing a significant blood volume to traverse the pulmonary vasculature without gas exchange. Some blood may pass through AV shunts, bypassing the alveoli and preventing gas exchange in these blood cells. Additionally, an increase in the thickness of the pulmonary capillary walls has been noted, leading to compromised gas diffusion. These pulmonary vasodilation processes, AV shunts, and impaired gas diffusion contribute to a ventilation-perfusion mismatch, resulting in an elevated alveolar-arterial gradient and hypoxemia. The most pronounced pulmonary vasodilation occurs in the lower regions of the lungs, manifesting symptoms such as platypnea and orthodeoxia commonly associated with HPS [20]. The proposed cause of hypoxemia is reduced oxygen diffusion across the dilated vessels, coupled with a shortened intrapulmonary blood transit time. This decreased transit time is attributed to the low vascular resistance in the intrapulmonary dilatations and the hyperdynamic circulation commonly observed in liver disease. As a result, there is no true shunt, and supplementing oxygen can significantly enhance PaO2 (partial pressure of arterial oxygen). Additionally, individuals with HPS exhibit reduced hypoxic pulmonary vascular constriction. Recent evidence indicates that intrapulmonary vascular dilatations manifest in two distinct patterns: Type I lesions, characterized by widespread pulmonary vascular dilatations with a positive PaO2 response to 100% oxygen; and Type II lesions, which involve more discrete, localized dilatations and show a poor response to oxygen [21,22].

Clinical features

HPS is a disease characterized by significant decreases in the arterial oxygenation of the lungs due to an underlying liver pathology. Therefore, it is significant to elucidate the clinical findings of a patient suffering from the condition. The early stages of the disease are mainly asymptomatic but may manifest as insidious respiratory complaints and findings associated with chronic liver disease [22]. Having a firm understanding of the clinical symptomatology associated with chronic liver disease is crucial [1,23]. Chronic liver disease findings include the development of esophageal varices, caput medusae, ascites, hepatic encephalopathy, and evidence of hypoestrogenism (spider naevi, gynecomastia, palmar erythema) [24]. The clinical picture involves non-specific physical examination findings, notably dyspnea on rest or exertion, the most common complaint [25]. Platypnea is a unique type of breathlessness characterized as the paradoxical shortness of breath exacerbated by sitting upright and improved by lying supine. This is paired with orthodoxia, low blood oxygen levels that typically worsen when the individual is upright due to vasodilation in the lower parts of the lungs and the increased blood flow through these areas when sitting up. These characteristic findings strongly suggest HPS but are absent in most patients, limiting their diagnostic value [26]. Pathologic respiratory and cardiac sounds are usually absent in HPS patients [1]. Worsening orthodeoxia-hypoxemia also suggests progressive disease and indicates developing arteriovenous malformation (AVM) [27]. The cornerstone in the clinical assessment of HPS is identifying the clinical symptomology associated with chronic liver disease. An existing diagnosis of longstanding portal hypertension, chronic viral hepatitis, or substance-induced liver cirrhosis is linked to a final diagnosis of HPS [28]. This can include generalized non-specific symptoms of weakness, fatigue, and anorexia or system-specific clinical manifestations (Table 1) [1,23,27-29]. Interestingly, the presence of spider naevi has been associated with HPS in multiple studies and has been attributed to being a significant sensitive finding [23,30].

**Table 1:** A Review of System-Specific Manifestations of Chronic Liver Disease References: [1,23,27-29]

System	Manifestations			
Generalized	Weakness, Fatigue, Anorexia, Weight Loss			
Dermal	Pruritus, Jaundice, Telangiectasia, Spider Angiomas, Palmar Erythema, Digital Clubbing, Terry Nails, Hypertrophic Osteoarthropathy			
Oral	Lacquered Lips, Smooth Red Tongue			
Abdominal	Nausea, Caput Medusae, Hepatomegaly, Splenomegaly, Ascites			
Endocrine	Male: Gynecomastia, Hypogonadism/Testicular Atrophy, Decreased Body Hair Female: Amenorrhea			
Vascular	GI or Esophageal Bleeding, Varices, Petechiae, and Purport			

Moreover, respiratory symptoms are frequently observed in cirrhosis individuals due to poor physical condition, smoking, ascites, and underlying lung problems [31]. This can make it challenging to diagnose HPS and can delay the diagnosis. Another important parameter that consistently remains abnormal in these patients is the decrease in the diffusion capacity of carbon monoxide (DLCO) [32]. It is important to be aware of the other possible differential diagnoses, most importantly portopulmonary hypertension (PPH) and hereditary hemorrhagic telangiectasia (HHT). PPH is characterized by elevated pulmonary pressure associated with chronic liver disease, where the resting pulmonary pressure is 25 mmHg and exceeds 30 mmHg during exercise. The development and progression of the disease involve unclear mechanisms, including pulmonary arteriopathy that leads to the obliteration of pulmonary vasculature. Mutations in the bone morphogenetic protein receptor type 2 (BMPR2) protein are implicated, but the exact mechanism, involving a hyperdynamic circulation, remains poorly understood [33]. Typical symptoms of PPH include breathlessness during exercise and more non-specific, which include fatigue, palpitations, and chest pain. A key cardiac sign includes a loud second tone of the pulmonary artery and a systolic heart murmur (tricuspid regurgitation) [34]. EKG shows signs of right ventricular hypertrophy and right atrial enlargement [33]. Diagnosis is confirmed through transthoracic echocardiography with the estimation of the right ventricular systolic pressure (>50 mmHg indicates the presence of PPH in >65% of patients) and by pulmonary artery catheterization to assess pulmonary arterial pressure, cardiac output, and pulmonary vascular resistance (PVR) [35].

Another important differential is HHT or Osler-Weber-Rendu syndrome, an autosomal dominant disorder that is associated with the pathologic formation of mucocutaneous telangiectasia and arterio-venous malformations in the lungs [33,36]. Furthermore, mutations in the ENG (chr.9) and ACVRL1 (chr.12) genes are responsible for the expression of endoglin and activin receptor-like kinase 1 (ACVRL1 or ALK1), respectively [36]. The elucidation of symptomology to differentiate from HPS is therefore significant, especially since, like HPS, HHT is a disease that can be relatively asymptomatic. Nevertheless, symptoms include the characteristic frequency of epistaxis. Dyspnea is also the most reported symptom, with pulmonary hypertension often resulting from the increased blood flow to the lungs. Significant AVMs in the pulmonary parenchyma can lead to symptoms such as exertional dyspnea, fatigue, and peripheral edema [37]. Hemorrhagic pulmonary AVMs also cause an increased risk of neurologic problems in adults [33]. Classic findings of pulmonary AVMs are similar to HPS, which include cyanosis, clubbing, and pulmonary bruit. HHT is also notorious for the presence of formation of emboli, including septic emboli, which has been seen to increase septic emboli and subsequent brain abscesses by 100-fold [38]. Dental work and poor dental hygiene should also be noted as significant risk factors. Another complication of HHT is stroke or transient ischemic attacks (TIAs) due to the paradoxical embolization of thrombus events via the pulmonary AVM formations [39]. Hemorrhage secondary to rupture of pulmonary AVMs can present as hemoptysis or hemothorax [40]. Furthermore, there is no relationship between liver disease and liver disease, so an absence of liver-disease clinical findings should be expected in HHT.

Diagnosis and screening

HPS is a rare complication that requires prompt recognition and staging to initiate appropriate management. Patients with HPS classically presents with dyspnea, platypnea, and orthodeoxia [41]. Patients with HPS additionally present with signs of liver dysfunction such as ascites, hepatomegaly, and jaundice [42]. These symptoms may be mistaken for other respiratory or liver conditions. Hence, diagnosis requires additional laboratory and radiological evaluations [43]. Screening for HPS is essential in individuals afflicted by chronic liver disease and who exhibit signs of dyspnea [6]. All patients eligible for liver transplantation should also be evaluated for HPS [44]. Screening for HPS initially requires using pulse oximetry as it can promptly confirm oxygen saturation in a patient [45].

Any patient displaying an oxygen saturation level below the threshold of 96% should receive an arterial blood gas (ABG) analysis to assess for the potential presence of underlying hypoxemia [46]. It is recommended for the ABG to be conducted with the patient in an upright position as HPS patients frequently present with orthodeoxia. Hypoxemia has an alveolar-arterial (A-a) gradient of more than 15 mmHg or a PaO2 of less than 80 mmHg [23,47]. Once it is confirmed that the patient is hypoxic, it is necessary to look for signs of intrapulmonary vasodilation (IPVD) [48]. IPVDs are a hallmark of HPS and must be verified to establish the diagnosis [49]. IPVDs are evaluated using a TTCE [49]. This procedure requires the creation of microbubbles using a saline syringe and injection into a selected peripheral vein [50]. A negative result of this test would show the microbubbles being absorbed in the pulmonary capillary bed and thus would not show up in the left atrium [51]. A positive outcome suggesting the existence of IPVDs would manifest as a delayed occurrence of the microbubbles within the left heart, specifically spanning three or more cardiac cycles after injection [51]. It is essential to distinguish the causes of the microbubbles seen in the left heart, as intracardiac shunts can produce a similar outcome [52]. Hence, an echocardiogram differentiates between IPDVs and intracardiac shunts. Another method of finding IPVDs is using a 99m Technetium-labeled macroaggregated albumin (Tc-MAA) lung perfusion scan. However, it is not preferred due to its low sensitivity (20-96%) compared to TTCE [53].

Staging of HPS is based on several criteria, which involve both laboratory and clinical findings (Table 2) [54]. HPS staging is based on arterial hypoxemia and the degree of intrapulmonary shunting. These variables categorize the patients into three stages: mild, moderate, or severe [55]. A patient with mild HPS typically has their PaO2 more than or equal to 80mmHg on room air [8]. Additionally, the shunt fraction calculated in mild HPS is typically less than 20%. Moderate HPS patients exhibit a PaO2 of more than 60 mmHg but less than 80 mmHg. The intrapulmonary shunt degree is generally between 20% and 30%. For severe HPS patients, the PaO2 is less than 60 mmHg, and the intrapulmonary shunting exceeds 30% [10].

**Table 2:** Hepatopulmonary Syndrome Severity Grading Reference: [54] PaO2: partial pressure of arterial oxygen

Hepatopulmonary Syndrome Severity Grading		PaO2 Levels (Arterial Oxygen Tension)		Arterial-Alveolar Oxygen Gradient (A-aO2)	
Mild		PaO2 ≥ 80 mmHg (≥ 10.7 kPa)		A-aO2 ≥ 15 mmHg (room air)	
Moderate		PaO2 ≥ 60 mmHg to < 80 mmHg (≥ 8 kPa and < 10.7 kPa)		A-aO2 ≥ 15 mmHg (room air)	
Severe		PaO2 ≥ 50 mmHg to < 60 mmHg (≥ 6.7 kPa and < 8 kPa)		A-aO2 ≥ 15 mmHg (room air)	
Very Severe		PaO2 < 50 mmHg (< 6.7 kPa)		A-aO2 ≥ 15 mmHg (room air)	
		PaO2 < 300 mmHg (40 kPa)		A-aO2 ≥ 15 mmHg (100% oxygen)	

HPS can be classified using angiographic patterns seen when pulmonary arteriography is conducted in patients with severe respiratory depression. Two types of HPS angiographic patterns have been documented. Type 1 findings show diffuse, normal vessels or fine, diffuse spidery vascular abnormalities [27]. In contrast, type 2 reports focal AV communications. In advanced cases of both types 1-2, the patient would find it difficult to breathe even with oxygen (Table 3) [27,56].

**Table 3:** Comprehensive Classification of Hepatopulmonary Syndrome Based on the Specific Radiological Findings and Patterns Divided into two types of patterns, this classification also provides a quantitative perspective on the prevalence of the common radiological findings [27,56].

Radiological Patterns of Hepatopulmonary Syndrome		Radiologic Description		Proportion of Total Cases	
Type 1		• Distal vascular dilatation accompanied by subpleural telangiectasia. • Its distinctive features encompass numerous subpleural vessels, slightly dilated, exhibiting atypical tapering, and extending to the pleural surface.		86%	
Type 2		• Identified as discrete arteriovenous malformations in angiographic images and the presence of nodular dilatation in peripheral pulmonary vessels.		14%	

Treatment

The only form of definitive treatment for HPS is a complete liver transplant, which leads to a total reversal of HPS maladaptive changes and provides the best overall long-term prognosis [1,8,57]. Multiple studies have demonstrated near-complete resolution of HPS-pathological symptoms following liver transplantation [1,57]. Initially, there is a rapid improvement of diagnostic PaO2 and A-a gradient within the first three months due to reduced pathological hepatocellular-induced IPVD [1,8,29,58]. This is followed by the resolution of hypoxemia within 6-12 months [8], and the reversal of orthodeoxia and platypnea within a year [1,8,23,57]. Definitive reversal of anomalous intrapulmonary vascular shunts is the last to resolve, noted in the literature to occur between 12 and 24 months following transplant [1,23,29,41,57,58].

The primary management strategy for HPS involves supportive symptomatic management. This involves supportive supplemental oxygen to reduce hypoxemia, maintain adequate oxygenation, and improve overall exercise tolerance and quality of life [1,23]. However, this remains only to manage superficial clinical symptoms and acts as a possible delay for disease progression but does not treat the underlying pathology [1]. Nonsurgical pharmacological treatment modalities have demonstrated few short-term symptomatic improvements in pulmonary functioning but none with notable long-term clinically significant benefits [1,23,57]. Transjugular intrahepatic portosystemic shunt (TIPS) procedure has had limited success in the early resolution of clinical symptoms of HPS. However, initial data suggests worsening respiratory clinical outcomes for HPS patients in the subacute period post-IPS procedure [1,8,23,29,51]. The proposed pathological mechanism involves a hyperkinetic circulatory state introduced by the TIPS procedure that aggravates intrapulmonary vascular shunts and further exacerbates IPVD [1,29]. In a clinical context, this condition presents as a swift deterioration in oxygen levels, increased difficulty in breathing, and orthodeoxia occurring within the first six months after the procedure.

Additionally, there is a suggestion of pulmonary arterial coil embolization as a less invasive surgical intervention aimed at addressing more pronounced anomalous intrapulmonary vascular shunts and alleviating severe clinical symptoms. However, this is only meant to serve as a temporizing measure and provides limited benefit in long-term HPS outcomes without definitive transplantation [29,57]. Published literature has demonstrated immediate improvement of clinical symptoms following embolization but eventual recurrence of intrapulmonary vascular shunts due to chronic IPVD [1,57].

## Conclusions

HPS is the abnormally dilated blood vessels and shunts within the lungs in the setting of portal hypertension, leading to impaired oxygenation, hypoxemia, and a complex immunomodulatory pathological change to the lungs. Both radiological evaluation and contrast-enhanced echocardiography are used in disease grading and classification along with the use of clinical markers indicating impaired oxygenation. Liver transplantation remains the definitive treatment modality based on the model for end-stage liver disease (MELD) policy. This review offers a succinct overview of the epidemiology, pathophysiology, causes, diagnostic approaches, and treatment options for HPS. Its goal is to enhance comprehension among clinicians and strengthen their capacity for making informed clinical decisions.

## References

[1] Bansal K, Gore M, Mittal S (2022). Hepatopulmonary syndrome. StatPearls [Internet].

[2] Rochon ER, Krowka MJ, Bartolome S (2020). BMP9/10 in pulmonary vascular complications of liver disease. Am J Respir Crit Care Med.

[3] Schenk P, Fuhrmann V, Madl C, Funk G, Lehr S, Kandel O, Müller C (2002). Hepatopulmonary syndrome: prevalence and predictive value of various cut offs for arterial oxygenation and their clinical consequences. Gut.

[4] Kennedy TC, Knudson RJ (1977). Exercise-aggravated hypoxemia and orthodeoxia in cirrhosis. Chest.

[5] Rodríguez-Roisin R, Agustí AG, Roca J (1992). The hepatopulmonary syndrome: new name, old complexities. Thorax.

[6] Younis I, Sarwar S, Butt Z (2015). Clinical characteristics, predictors, and survival. Ann Hepatol.

[7] Kim BJ, Lee SC, Park SW (2004). Characteristics and prevalence of intrapulmonary shunt detected by contrast echocardiography with harmonic imaging in liver transplant candidates. Am J Cardiol.

[8] Pascasio JM, Grilo I, López-Pardo FJ (2014). Prevalence and severity of hepatopulmonary syndrome and its influence on survival in cirrhotic patients evaluated for liver transplantation. Am J Transplant.

[9] Krowka MJ, Wiseman GA, Burnett OL, Spivey JR, Therneau T, Porayko MK, Wiesner RH (2000). Hepatopulmonary syndrome: a prospective study of relationships between severity of liver disease, PaO(2) response to 100% oxygen, and brain uptake after (99m)Tc MAA lung scanning. Chest.

[10] Deberaldini M, Arcanjo AB, Melo E (2008). Hepatopulmonary syndrome: morbidity and survival after liver transplantation. Transplant Proc.

[11] Dahiya DS, Kichloo A, Shaka H (2021). Hepatopulmonary syndrome: a nationwide analysis of epidemiological trends and outcomes from 2012 to 2018. Gastroenterology Res.

[12] Fallon MB, Krowka MJ, Brown RS (2008). Impact of hepatopulmonary syndrome on quality of life and survival in liver transplant candidates. Gastroenterology.

[13] Noli K, Solomon M, Golding F, Charron M, Ling SC (2008). Prevalence of hepatopulmonary syndrome in children. Pediatrics.

[14] Tumgor G, Arikan C, Yuksekkaya HA (2008). Childhood cirrhosis, hepatopulmonary syndrome and liver transplantation. Pediatr Transplant.

[15] Grace JA, Angus PW (2013). Hepatopulmonary syndrome: update on recent advances in pathophysiology, investigation, and treatment. J Gastroenterol Hepatol.

[16] Sato K, Oka M, Hasunuma K, Ohnishi M, Sato K, Kira S (1995). Effects of separate and combined ETA and ETB blockade on ET-1-induced constriction in perfused rat lungs. Am J Physiol.

[17] Carter EP, Hartsfield CL, Miyazono M, Jakkula M, Morris KG Jr, McMurtry IF (2002). Regulation of heme oxygenase-1 by nitric oxide during hepatopulmonary syndrome. Am J Physiol Lung Cell Mol Physiol.

[18] Caramelo C, Fernández-Gallardo S, Santos JC, Iñarrea P, Sánchez-Crespo M, López-Novoa JM, Hernando L (1987). Increased levels of platelet-activating factor in blood from patients with cirrhosis of the liver. Eur J Clin Invest.

[19] Schraufnagel DE, Kay JM (1996). Structural and pathologic changes in the lung vasculature in chronic liver disease. Clin Chest Med.

[20] Rodríguez-Roisin R, Krowka MJ, Agustí A (2018). Hepatopulmonary disorders: gas exchange and vascular manifestations in chronic liver disease. Compr Physiol.

[21] Naeije R, Hallemans R, Mols P, Melot C (1981). Hypoxic pulmonary vasoconstriction in liver cirrhosis. Chest.

[22] Varghese J, Ilias-Basha H, Dhanasekaran R (2007). Hepatopulmonary syndrome - past to present. Ann Hepatol.

[23] Rodríguez-Roisin R, Krowka MJ (2008). Hepatopulmonary syndrome - a liver-induced lung vascular disorder. N Engl J Med.

[24] Sharma A, Nagalli S (2023). Chronic liver disease. StatPearls [Internet].

[25] Ghittoni G, Valentini G, Spada C (2003). Hepatopulmonary syndrome. A review of the literature. Panminerva Med.

[26] Fallon MB, Abrams GA (2000). Hepatopulmonary syndrome. Curr Gastroenterol Rep.

[27] Krowka MJ, Dickson ER, Wiesner RH, Krom RA, Atkinson B, Cortese DA (1992). A prospective study of pulmonary function and gas exchange following liver transplantation. Chest.

[28] Lange PA, Stoller JK (1995). The hepatopulmonary syndrome. Ann Intern Med.

[29] Fritz JS, Fallon MB, Kawut SM (2013). Pulmonary vascular complications of liver disease. Am J Respir Crit Care Med.

[30] Wang YW, Lin HC (2005). Recent advances in hepatopulmonary syndrome. J Chin Med Assoc.

[31] Crapo RO, Jensen RL, Hegewald M, Tashkin DP (1999). Arterial blood gas reference values for sea level and an altitude of 1,400 meters. Am J Respir Crit Care Med.

[32] Martínez GP, Barberà JA, Visa J (2001). Hepatopulmonary syndrome in candidates for liver transplantation. J Hepatol.

[33] Krynytska I, Marushchak M, Mikolenko A, Bob A, Smachylo I, Radetska L, Sopel O (2017). Differential diagnosis of hepatopulmonary syndrome (HPS): portopulmonary hypertension (PPH) and hereditary hemorrhagic telangiectasia (HHT). Bosn J Basic Med Sci.

[34] Hoeper MM, Krowka MJ, Strassburg CP (2004). Portopulmonary hypertension and hepatopulmonary syndrome. Lancet.

[35] Ramsay M (2010). Portopulmonary hypertension and right heart failure in patients with cirrhosis. Curr Opin Anaesthesiol.

[36] Sharathkumar AA, Shapiro A (2008). Hereditary haemorrhagic telangiectasia. Haemophilia.

[37] Faughnan ME, Granton JT, Young LH (2009). The pulmonary vascular complications of hereditary haemorrhagic telangiectasia. Eur Respir J.

[38] Kjeldsen AD, Tørring PM, Nissen H, Andersen PE (2014). Cerebral abscesses among Danish patients with hereditary haemorrhagic telangiectasia. Acta Neurol Scand.

[39] White RI Jr, Lynch-Nyhan A, Terry P (1988). Pulmonary arteriovenous malformations: techniques and long-term outcome of embolotherapy. Radiology.

[40] Ference BA, Shannon TM, White RI Jr, Zawin M, Burdge CM (1994). Life-threatening pulmonary hemorrhage with pulmonary arteriovenous malformations and hereditary hemorrhagic telangiectasia. Chest.

[41] Gómez FP, Martínez-Pallí G, Barberà JA, Roca J, Navasa M, Rodríguez-Roisin R (2004). Gas exchange mechanism of orthodeoxia in hepatopulmonary syndrome. Hepatology.

[42] Hemprich U, Papadakos PJ, Lachmann B (2010). Respiratory failure and hypoxemia in the cirrhotic patient including hepatopulmonary syndrome. Curr Opin Anaesthesiol.

[43] Kawut SM, Krowka MJ, Forde KA (2022). Impact of hepatopulmonary syndrome in liver transplantation candidates and the role of angiogenesis. Eur Respir J.

[44] Bommena S, Gerkin RD, Agarwal S, Raevens S, Glassberg MK, Fallon MB (2021). Diagnosis of hepatopulmonary syndrome in a large integrated health system. Clin Gastroenterol Hepatol.

[45] Deibert P, Allgaier HP, Loesch S (2006). Hepatopulmonary syndrome in patients with chronic liver disease: role of pulse oximetry. BMC Gastroenterol.

[46] Voiosu A, Voiosu T, Stănescu CM (2013). Novel predictors of intrapulmonary vascular dilatations in cirrhosis: extending the role of pulse oximetry and echocardiography. Acta Gastroenterol Belg.

[47] Krowka MJ, Dickson ER, Cortese DA (1993). Hepatopulmonary syndrome. Clinical observations and lack of therapeutic response to somatostatin analogue. Chest.

[48] Forde KA, Fallon MB, Krowka MJ (2019). Pulse oximetry is insensitive for detection of hepatopulmonary syndrome in patients evaluated for liver transplantation. Hepatology.

[49] Abrams GA, Jaffe CC, Hoffer PB, Binder HJ, Fallon MB (1995). Diagnostic utility of contrast echocardiography and lung perfusion scan in patients with hepatopulmonary syndrome. Gastroenterology.

[50] Grilo I, Pascasio JM, Tirado JL (2017). The utility of the macro-aggregated albumin lung perfusion scan in the diagnosis and prognosis of hepatopulmonary syndrome in cirrhotic patients candidates for liver transplantation. Rev Esp Enferm Dig.

[51] Tonelli AR, Naal T, Dakkak W, Park MM, Dweik RA, Stoller JK (2017). Assessing the kinetics of microbubble appearance in cirrhotic patients using transthoracic saline contrast-enhanced echocardiography. Echocardiography.

[52] Alipour Z, Armin A, Mohamadi S, Tabib SM, Azizmohammadi Z, Gholamrezanezhad A, Assadi M (2020). Hepatopulmonary syndrome with right-to-left shunt in cirrhotic patients using macro-aggregated albumin lung perfusion scan: comparison with contrast echocardiography and association with clinical data. Mol Imaging Radionucl Ther.

[53] El-Shabrawi MH, Omran S, Wageeh S (2010). (99m)Technetium-macroaggregated albumin perfusion lung scan versus contrast enhanced echocardiography in the diagnosis of the hepatopulmonary syndrome in children with chronic liver disease. Eur J Gastroenterol Hepatol.

[54] Rodríguez-Roisin R, Krowka MJ, Hervé P, Fallon MB (2004). Pulmonary-hepatic vascular disorders (PHD). Eur Respir J.

[55] Fussner LA, Krowka MJ (2016). Current approach to the diagnosis and management of portopulmonary hypertension. Curr Gastroenterol Rep.

[56] Kim YK, Kim Y, Shim SS (2009). Thoracic complications of liver cirrhosis: radiologic findings. Radiographics.

[57] Powers KA, Dhamoon AS (2023). Physiology, pulmonary ventilation and perfusion. StatPearls [Internet].

[58] Cheng TO (2002). Mechanisms of platypnea-orthodeoxia: what causes water to flow uphill?. Circulation.

